# Netrin-1/DCC Signaling Differentially Regulates the Migration of Pax7, Nkx6.1, Irx2, Otp, and Otx2 Cell Populations in the Developing Interpeduncular Nucleus

**DOI:** 10.3389/fcell.2020.588851

**Published:** 2020-10-20

**Authors:** Isabel M. García-Guillén, Antonia Alonso, Nicanor Morales-Delgado, Belén Andrés, Luis Puelles, Guillermina López-Bendito, Faustino Marín, Pilar Aroca

**Affiliations:** ^1^Department of Human Anatomy and Psychobiology, School of Medicine, University of Murcia, Murcia, Spain; ^2^Biomedical Research Institute of Murcia (IMIB-Arrixaca), Murcia, Spain; ^3^Department of Histology and Anatomy, School of Medicine, Miguel Hernández University, Alicante, Spain; ^4^Instituto de Neurociencias de Alicante, CSIC, Universidad Miguel Hernández, Alicante, Spain

**Keywords:** neuronal tangential migration, axon guidance, Netrin1, DCC, transcription factors, rhombomeres, chemoattractant molecules, interpeduncular nucleus (IPN)

## Abstract

The interpeduncular nucleus (IPN) is a hindbrain structure formed by three main subdivisions, the prodromal (Pro) domain located at the isthmus (Ist), and the rostral and caudal interpeduncular domains (IPR, IPC) within rhombomere 1 (r1). Various cell populations can be detected in the IPN through the expression of the *Nkx6.1*, *Otp*, *Otx2*, *Pax7*, and/or *Irx2* transcription factors. These cell populations follow independent dorsoventral tangential and radial migratory routes targeting the ventral paramedian region of Ist and r1. Here we set out to examine the influence of the Netrin-1/DCC pathway on these migrations, since it is known to regulate other processes of neuronal migration in the brain. To this end, we analyzed IPN development in late gestational wild-type and DCC^–/–^ mice, using mainly *in situ* hybridization (ISH) to identify the cells expressing each of the aforementioned genes. We found that the migration of *Nkx6.1*^+^ and *Irx2*^+^ cells into the Pro domain was strongly disrupted by the loss of DCC, as occurred with the migration of *Pax7*^+^, *Irx2*^+^, and *Otp*^+^ cells that would normally form the IPR. In addition, there was mild impairment of the migration of the *Pax7*^+^ and *Otx2*^+^ cells that form the IPC. These results demonstrate that the Netrin-1/DCC signaling pathway is involved in the migration of most of the IPN populations, mainly affecting those of the Pro and IPR domains of this nucleus. There are psychiatric disorders that involve the medial habenula (mHb)-IPN system, so that this experimental model could provide a basis to study their neurodevelopmental etiology.

## Introduction

Neuronal migration is an important feature of brain development, in some cases involving the displacement of considerable cell populations toward different coordinates. In addition to the classic examples of migration, such as that in the precerebellar nuclei (Bloch-Gallego et al., 2005), recent data has shown that complex migratory events contribute to the formation of the interpeduncular nucleus (IPN) (Lorente-Cánovas et al., 2012; Moreno-Bravo et al., 2014; Ruiz-Reig et al., 2019). In vertebrates, the IPN is a complex hindbrain structure and it is the principal target of the retroflex tract that comes from the medial habenula (mHb) (Herkenham and Nauta, 1979; Contestabile and Flumerfelt, 1981). In behavioral and functional studies, the mHb-IPN pathway has been implicated in learning and memory, affective states and mood-related psychiatric conditions (Klemm, 2004; Hikosaka, 2010). However, the development of the IPN has only recently been studied, in particular in the chick (Lorente-Cánovas et al., 2012) and mouse (Moreno-Bravo et al., 2014; Ruiz-Reig et al., 2019).

Our previous experimental study in the chick demonstrated that the IPN is formed in the rostral (prepontine) hindbrain, containing cells from both alar and basal populations (Lorente-Cánovas et al., 2012). Each of the IPN populations follows a specific dorsoventral migratory trajectory from its original periventricular position, ultimately converging at a more medial site and settling down in a sub-pial location across the median floor plate of the isthmus (Ist) and hindbrain rhombomere 1 (r1) (Lorente-Cánovas et al., 2012; Moreno-Bravo et al., 2014). At the end of this process, the so-called rostral (IPR) and caudal (IPC) domains of the IPN lie in the rostral and caudal parts of r1 (r1-r, r1-c), respectively, while the smaller rostral prodromal (Pro) domain is located in the isthmic region. In terms of the molecular regionalization of this nucleus, we identified at least four distinct neuronal populations that expressed the *Pax7* (alar plate origin), and *Nkx6.1*, *Otp* or *Otx2* (distinct basal plate origins) transcription factors.

Based on their morphology, migratory routes, location and gene expression, the same neuronal populations that form the chick IPN also appear to generate the mouse IPN (García-Guillén et al., unpublished; Moreno-Bravo et al., 2014; Ruiz-Reig et al., 2019). Moreover, Otx2 activity is required for the tangential migration of the IPC cells that normally express this gene (Ruiz-Reig et al., 2019), and a conditional mutant of *Shh*, the gene responsible for the formation of the floorplate, alters the migration of the *Pax7*^+^, *Nkx6.1*^+^, *Otp*^+^, and *Otx2*^+^ cells that would normally form the distinct parts of the IPN (Moreno-Bravo et al., 2014). These earlier studies showed that the IPN emerges as a plurisegmental and heterogeneous alar/basal formation whose correct development depends on the proper migration and positioning of several neuronal populations. However, the molecular mechanisms underlying these independent migratory events are still largely unknown, other than the need for SHH at the floor plate (Moreno-Bravo et al., 2014).

One of the molecular mechanisms that regulates neuronal migration in the brain is the Netrin-1/DCC (Deleted in Colorectal Cancer) pathway (Bashaw and Klein, 2010; Marín et al., 2010; Evsyukova et al., 2013). Netrin-1 is expressed in the floorplate of the central nervous system and it acts as an attractive molecule in conjunction with its receptor, DCC (Fearon et al., 1990; Hedrick et al., 1994; Keino-Masu et al., 1996). Floorplate-derived Netrin-1/DCC signaling is known to steer the migration of several neuronal populations in the hindbrain. Indeed, it is required for the dorsoventral tangential migration of noradrenergic neurons of the locus coeruleus (Shi et al., 2008) and for the migration of precerebellar rhombic lip neurons (Alcántara et al., 2000; de Diego et al., 2002; Bloch-Gallego et al., 2005), including inferior olivary neurons (Bloch-Gallego et al., 2005; Marcos et al., 2009) and neurons of the basilar pontine nuclei (Kratochwil et al., 2017). In terms of the IPN, *Netrin-1* is expressed in the floor plate of the rostral hindbrain where this nucleus develops, while *Dcc* is expressed by the migrating *Otx2*^+^ cells that enter the IPC (Ruiz-Reig et al., 2019).

Taking all this into account, we have studied the possible functional role of the Netrin-1/DCC pathway in regulating the dorsoventral tangential and/or radial migrations of the diverse IPN populations. To this end, we analyzed IPN development in an experimental mouse model devoid of DCC activity, demonstrating that practically all the selective cell migration to the IPN is altered in these mice.

## Materials and Methods

### Animals and Embryo Processing

**FIGURE 1:**
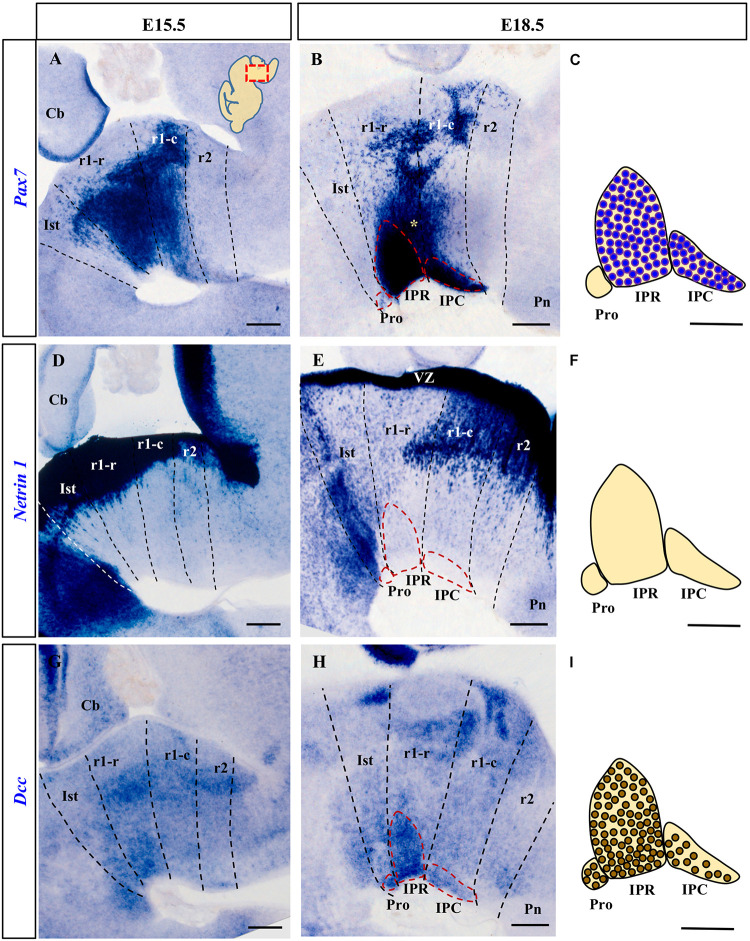
Comparison of *Pax7*, *Netrin-1* and *Dcc* rostral hindbrain expression in sections from E15.5 and E18.5 mouse embryos processed for ISH (see section Materials and Methods). In this and the other figures, the images are from parasagittal sections centered on the IPN in the rostral hindbrain. In each image the rostral end is to the left. The dashed lines represent the intersegmental boundaries delimiting the Ist, r1-r, r1-c and r2. **(A,B)** *Pax7* is a marker for the majority of cells of the developing IPN. At E15.5 the *Pax7*^+^ cells are migrating in the mantle layer, while at E18.5 they are forming the IPR and the IPC. **(D,E)** *Netrin-1* is expressed strongly by the floor plate in the Ist and r1 where the IPN develops from the early (data not shown) to later stages, at E15.5 and E18.5. **(G,H)** At E15.5, *Dcc* is expressed by IPN neurons migrating to the IPN to become part of the three portions of the nucleus (Pro, IPR, and IPC) at E18.5. **(C,F,I)** Scheme of sagittal sections of the IPN at E18.5, from top to bottom representing: the expression of *Pax7* by cells in the IPR and IPC, the absence of *Netrin-1* expression in the nucleus, and the expression of the netrin receptor *Dcc* by IPR, IPC and (unlike *Pax7*) Pro cells. Pro, prodromal; IPR, interpeduncular rostral; IPC, interpeduncular caudal; Ist, isthmus; r1-r, rhombomere 1 rostral; r1-c, rhombomere 1 caudal; r2, rhombomere 2; Pn, pontine nuclei; VZ, ventricular zone; Cb, cerebellum. **(A,B,D,E,G,H)** scale bars = 200 μm, **(C,F,I)** scale bars = 50 μm.

All mice were maintained according to the European Union guidelines (2010/63/EU) and Spanish law (Royal Decree 53/2013 and Royal Decree 1386/2018) regarding the care and handling of research animals. All procedures were performed according to protocols approved by the University of Murcia Ethical Committee for Animal Experimentation. Swiss albino mice bred at the University of Murcia were used to analyze gene expression during development (Figure 1). The *Dcc* knock-out mice (Fazeli et al., 1997) were generated and kindly provided by Marc Tessier-Lavigne (The Rockefeller University; New York, United States).

In all cases, the day of vaginal post-coital plug detection was regarded as embryonic day 0.5 (E0.5). E15.5 and E18.5 embryos of undetermined sex were extracted from the pregnant dams after sacrifice, genotyped and fixed overnight in 4% paraformaldehyde (PFA) prepared in phosphate-buffered saline (PBS, pH 7.4). For the studies of the transgenic mice, embryos from the same litter were matched in pairs of +/+ and −/− mice for each experiment. The embryo’s brain was dissected out, post-fixed at 4°C in the PFA for 24 h (E15.5 embryos) or 48 h (E18.5 embryos), and then washed in PBS. The brains were then embedded in 4% agarose in PBS, and sagittal vibratome sections (80 μm thick) were subsequently processed for *in situ* hybridization (ISH) and in some cases, followed by immunohistochemistry (IHC).

### RNA Probes

Digoxigenin-labeled riboprobes were synthesized from cDNA constructs kindly provided by M. Tessier-Lavigne (*Netrin-1* and *Dcc*: Rockefeller University, NY, United States), A. Simeone (*Otp* and *Otx2*: Inst. Genetics and Biophysics, Naples, Italy) and O. Marín (*Nkx6.1*: King’s College London, United Kingdom). A *Pax7* cDNA clone (Riken 5330440B08, NCBI accession number AK133693) was obtained from Source BioScience (Nottingham, United Kingdom) and an *Irx2* cDNA clone (MGC 27958, IMAGE 3592499, NCBI accession number BC029750) from ImaGenes GmbH (Berlin, Germany).

### *In situ* Hybridization and Immunohistochemistry

Floating sagittal sections were processed for ISH with digoxigenin-labeled antisense RNA probes using the protocol of Nieto et al. (1996) adapted for floating vibratome sections. To detect the hybridized probes, the sections were incubated overnight with alkaline phosphatase (AP)-conjugated anti-digoxigenin antibody, and the NBT/BCIP substrates were used to detect AP activity, producing a blue chromogenic precipitate. The sections were then washed in saline solution and post-fixed.

To combined IHC with ISH, the sections were incubated with a monoclonal mouse anti-Pax7 antibody (Developmental Studies Hybridoma Bank) after ISH, diluted 1:80 in Tris-buffered saline with 0.1% Tween 20 and 20% of sheep serum. The binding of this antibody was detected with a biotinylated goat anti-mouse IgG secondary antibody (Sigma) diluted 1:200 in Tris-buffered saline with 0.1% Tween 20 and 5% sheep serum and after several washes in Tris-buffered saline with 0.05% Tween 20, it was visualized with avidin-biotin-peroxidase (VECTASTAIN Elite ABC kit from Vector), following the manufacturer’s indications. Peroxidase was detected by standard protocols, using diaminobenzidine-tetrahydrochloride (DAB) and hydrogen peroxide as substrates to produce a brown chromogenic precipitate. Finally, the sections were mounted, dehydrated and then coverslipped with Eukitt. Digital photographs were taken on a Leica microscope (DMR HC) equipped with a Zeiss Axiovision digital camera.

## Results

We analyzed the expression of *Netrin-1*, *Dcc* and the genes indicated, characterizing the distinct IPN neuronal populations in the brain sections from both wild-type and DCC^–/–^ mice, situating them relative to the Ist, r1-r, r1-c and rhombomere 2 (r2) boundaries. We identified these boundaries based on the mapping of these regions and the expression data available in chick and mouse brains (Aroca and Puelles, 2005; Aroca et al., 2006; Lorente-Cánovas et al., 2012; Alonso et al., 2013; Watson et al., 2017).

### The Netrin-1 Receptor DCC Is Expressed in the Developing Interpeduncular Nucleus

We first analyzed *Netrin-1* and *Dcc* mRNA expression in the IPN territory at several stages of development, identified by the position of the migrated *Pax7*^+^ cells. According to previous studies in the chick (Lorente-Cánovas et al., 2012), the *Pax7*^+^ IPN population originates in the alar plate of the ventricular zone (VZ) throughout r1 (r1-r and r1-c), from where the cells first translocate tangentially into the mantle of the medial basal plate, and then, they migrate radially toward the pial surface. In the mouse, we found that the *Pax7*^+^ cells began to reach the median IPN locus at E15.5 (Figure 1A) and by E18.5, *Pax7* is expressed strongly by neurons in the IPR and IPC, but not by those in isthmic Pro (Figures 1B,C). In addition to the cells in the IPN, at this stage there was still a large population of cells that express *Pax7* migrating en route to that nucleus (yellow asterisk in Figure 1B). Other labeled populations remain stable in the periventricular stratum, probably corresponding to the prospective populations of the ventral tegmental and sphenoid nuclei (Moreno-Bravo et al., 2014). From early stages of development (E11.5 and E13.5), *Netrin-1* is intensely expressed in the VZ of the hindbrain floor plate, including that of Ist and r1 (data not shown). This VZ contains the cell bodies of the radial glia that constitute the main cell population in the floor plate. At stages E15.5 and E18.5 (Figures 1D,E), *Netrin-1* was also expressed by some neuron populations within the basal mantle of Ist and r1 but not in the IPN complex itself (Figures 1E,F).

In terms of the Netrin-1 receptor, *Dcc* was expressed in the VZ throughout the Ist and r1 at E11.5 and E13.5 (data not shown). At E15.5, there were numerous *Dcc*^+^ cells in the mantle layer of these segments and a population of *Dcc*^+^ neurons begin to arrive at the IPN locus (Figure 1G). By E18.5, the *Dcc*^+^ population in the IPN had increased markedly, distributed across its three rostro-caudal domains: Pro, IPR and IPC (Figures 1H,I). A stream of *Dcc*^+^ cells was also evident along the radial migratory pathway to the IPN.

Comparing the expression of *Dcc* and *Pax7*, there was a clear overlap in the expression of both markers in the IPR and IPC domains of the IPN, while the Pro domain was *Pax7*^–^ and *Dcc*^+^ (compare Figures 1B,C,H,I). These expression patterns complemented the distribution of *Otx2* cells within the IPC (Ruiz-Reig et al., 2019) and they were consistent with the possible regulation of the migration of the different components of the IPN by Netrin-1/DCC signaling, particularly given the uniform *Dcc* expression in the migrating IPN cells and the presence of *Netrin-1* in their target region, the rostral hindbrain floorplate.

### The Migration of Five Independent IPN Neuronal Populations Is Differentially Disrupted in DCC^–/–^ Embryos

Considering the aforementioned hypothesis, we evaluated the migration of the *Nkx6.1*^+^, *Irx2*^+^, *Pax7*^+^, *Otp*^+^, and *Otx2*^+^ IPN cell populations in E18.5 DCC^–/–^ and wild-type mice. *Irx2* was recently identified as a conspicuous marker for the IPN in a screening of genes expressed in this structure (García-Guillén et al., unpublished), while the other markers indicated have been characterized previously (Lorente-Cánovas et al., 2012; Moreno-Bravo et al., 2014; Ruiz-Reig et al., 2019).

**FIGURE 2:**
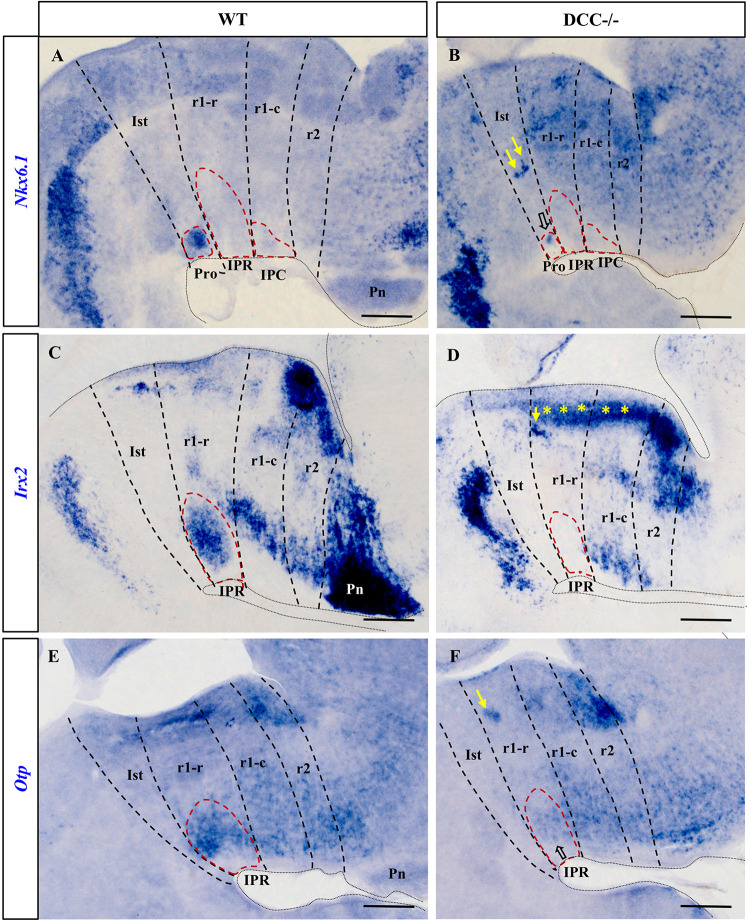
Comparison of *Nkx6.1*, *Irx2* and *Otp* expression in sections processed for ISH from wild-type and DCC^–/–^ brains at E18.5. **(A,C,E)** Sections of wild-type brains showing the expression of these genes. *Nkx6.1* **(A)** is expressed by the cells in the Pro, *Otp* **(E)** by cells in the IPR and *Irx2* **(C)** is expressed both in the Pro and the IPR, although *Irx2* expression in the Pro is not visualized here because this image corresponds to a medial sagittal section and the Pro lies more laterally. Along with **(C)**, the images **(D,E,F)** correspond to medial sagittal sections where the Pro and the IPC are absent. **(B,D,F)** Sections of DCC^–/–^ brains showing the respective expression of these genes. There are no *Irx2* cells in the IPR **(D)** or in the Pro (data not shown), whereas *Nkx6.1* **(B)** and *Otp* **(F)** are almost absent in the Pro and IPR respectively, with some cells that have reached their correct location in the IPN in the latter two cases (open arrows). In these three images, yellow arrows indicate patches of cells that have ceased to migrate toward the IPN, probably as a consequence of the absence of DCC. Yellow asterisks in **(D)** indicate an aberrant band of *Irx2*^+^ cells that appear not to have completed the migratory process through the mantle in DCC^–/–^ mice. Pro, prodromal; IPR, interpeduncular rostral; IPC, interpeduncular caudal; Ist, isthmus; r1-r, rhombomere 1 rostral; r1-c, rhombomere 1 caudal; r2, rhombomere 2; Pn, pontine nuclei. Scale bars = 200 μm.

In wild-type embryos, *Nkx6.1* expressing cells were only observed in the Pro domain in the isthmic region of the IPN (Figure 2A). This *Nkx6.1^+^* population was significantly smaller in DCC^–/–^ embryos, such that only a few cells reached their final location in the Pro domain (Figure 2B). In addition, anomalous ectopic patches of *Nxk6.1*^+^ cells were evident in the isthmic tegmentum (yellow arrows in Figure 2B), probably corresponding to disorientated cells from this population.

The *Irx2*^+^ population was distributed in the Pro and IPR in wild-type embryos (Figure 2C and data not shown), yet there were no *Irx2*^+^ cells in these two compartments of the DCC^–/–^ IPN (Figure 2D). Rather, such cells appeared to accumulate densely in the corresponding periventricular stratum, as well as in ectopic patches within the r1 tegmentum (yellow asterisks and yellow arrow in Figure 2D), reflecting their disturbed migration. *Irx2* is also a marker of the pontine nuclei, such that the disruption to the expression of this gene also reflects the failure of these nuclei to develop in DCC^–/–^ embryos. The effects on these structures may have been expected since Netrin-1/DCC signaling was demonstrated to regulate migration to the pontine nuclei (Alcántara et al., 2000).

Like the *Irx2*^+^expressing cells, *Otp*^+^ cells in wild-type embryos were mainly observed in the IPR (Figure 2E), whereas these cells were almost completely absent from this structure in DCC^–/–^ embryos (Figure 2F). We also observed a dense group of ectopic *Otp*^+^ cells in the r1 tegmental mantle of DCC^–/–^ embryos that may have had their migration arrested (yellow arrow in Figurr 2F).

**FIGURE 3:**
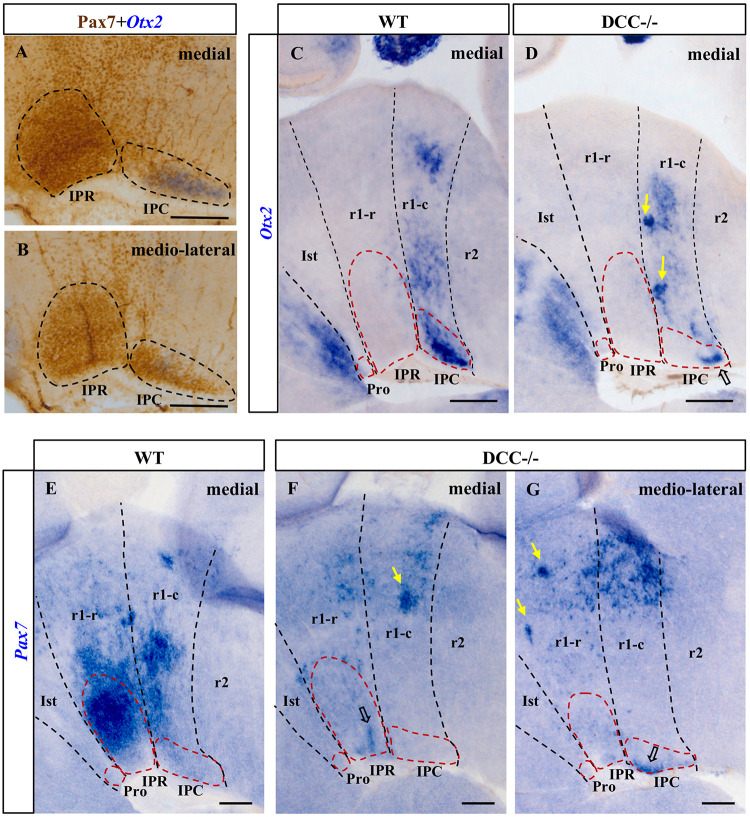
Comparison of the *Otx2* and *Pax7* expression in sections from E18.5 wild-type and DCC^–/–^ brains processed for ISH plus IHC **(A,B)** or ISH alone **(C–G)**. **(A,B)** Parasagittal sections of the IPN in an E18.5 wild-type brain after dual ISH-IHC showing the expression of Pax7 (IHC: brown precipitate) and *Otx2* (ISH: blue precipitate) in the IPN. While Pax7 is expressed in the entire IPR, there are two independent populations in the IPC: the *Otx2*^+^ population located in the most medial part of the IPC **(A)**, while the Pax7^+^ population is placed laterally to the *Otx2* population **(B)**. Therefore, the medio-lateral Pax7^+^ population embraces the medial *Otx2* population in the IPC. **(C,E)** Sections of wild-type brains showing the expression of these genes. *Otx2* is expressed by cells in the IPC (medially: **C**) while *Pax7* is expressed in the IPR (medially: **E)** and the IPC (medio-laterally: data not shown). We cannot see *Pax7* expression in the IPC in **(E)** as this image is a medial sagittal section, and the caudal expression of *Pax7* is observable more laterally. **(D,F,G)** Sections of DCC^–/–^ brains showing the expression of these genes. A large part of the *Otx2*^+^ population in the IPC is absent, although some cells have reached their correct location in the caudal IPC (open arrow in **D**). The rostral *Pax7* population has almost completely disappeared from the IPR, with only a few cells arriving at their destination (open arrow in **F**), whereas the caudal population seems to be less severely affected since more *Pax7*^+^ cells reach their correct location in the IPC (open arrow in **G**). In the latter three images, the yellow arrows point to patches of cells that appear to have halted their migration to the IPN as a consequence of the absence of DCC. Pro, prodromal; IPR, interpeduncular rostral; IPC, interpeduncular caudal; Ist, isthmus; r1-r, rhombomere 1 rostral; r1-c, rhombomere 1 caudal; r2, rhombomere 2. **(A,B)** scale bars = 50 μm, **(C,D,E,F,G)** scale bars = 200 μm.

**FIGURE 4:**
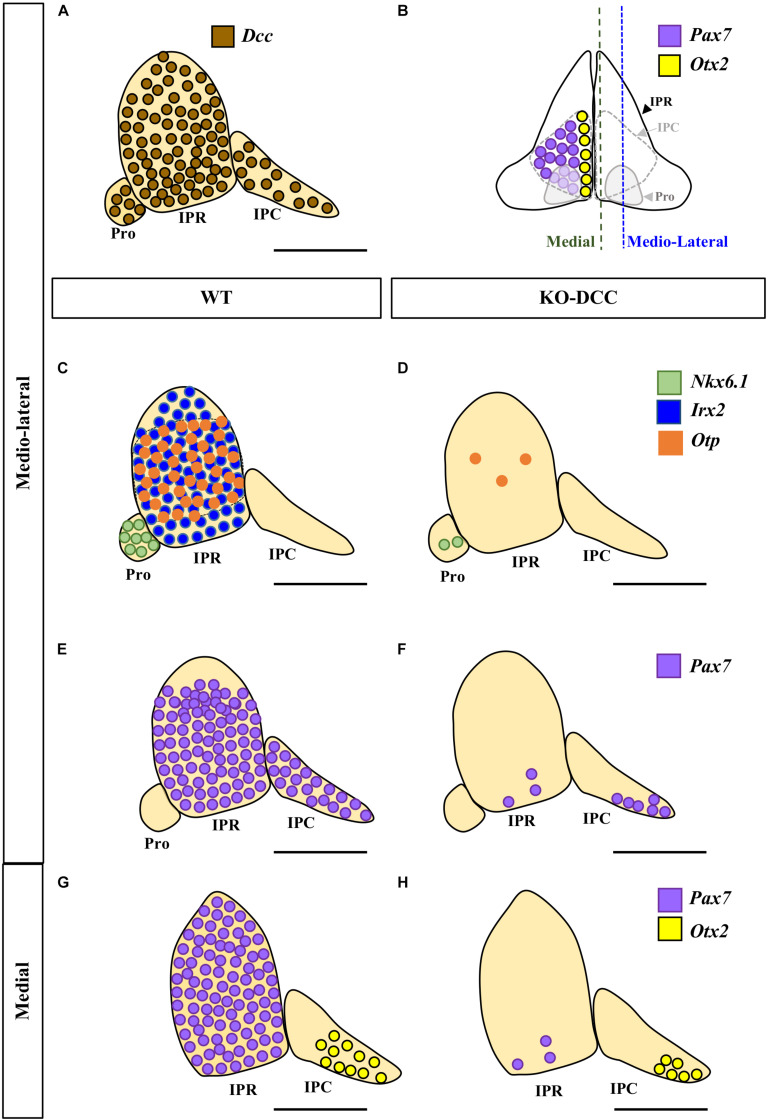
Comparison of the wild-type and DCC^–/–^ phenotypes of the IPN. **(A,C,E,G)** Sagittal diagrams showing the distribution of *Dcc* (brown circles), *Nkx6.1* (green circles), *Irx2* (blue circles), *Otp* (orange circles), *Pax7* (purple circles) and *Otx2* (yellow circles) expressing cells in the IPN. **(B)** Scheme of a frontal view of the IPN at E18.5 in which its sub-regions and the distribution of the caudal populations of the IPN are shown, with the *Pax7*^+^ population embracing the *Otx2*^+^ population. **(D,F,H)** Sagittal diagrams of the IPN in DCC^–/–^ brains following the aforementioned color codes. By comparing **(C)** and **(D)** we can see that the *Nkx6.1*^+^ population disappears almost entirely in the Pro of the DCC^–/–^ brains. Similarly, a large part of the *Otp*^+^ population disappears from the IPR while the *Irx2*^+^ population is completely absent in these mutants. The *Pax7*^+^ population of the IPR disappears almost completely (compare **E** to **F**, and **G** to **H**). However, the *Pax7*^+^ population in the IPC (located medio-laterally) is less affected (compare **E** to **F**) than the aforementioned rostral populations. Similarly, the *Otx2*^+^ population of the IPC (located medially) is less affected than the other populations of the IPN, although a large part of this population has also disappeared (compare **G** to **H**). Pro, prodromal; IPR, interpeduncular rostral; IPC, interpeduncular caudal. Scale bars = 50 μm.

To analyze the effect of Netrin-1/DCC signaling on the development of the *Otx2*^+^ and *Pax7*^+^ IPN populations, it must be borne in mind that *Pax7* is expressed extensively in the IPR, including the medial and lateral aspects of this structure (Figures 3A,B). By contrast, the most medial population in the IPC is *Otx2*^+^ (Figure 3A), with *Pax7*^+^ cells situated laterally (Figure 3B and diagram in Figure 4B). These patterns in mice are similar to those observed for the same genes in the chick IPN (Lorente-Cánovas et al., 2012).

The migration of *Pax7*^+^ cells into the medial aspect of the IPR domain almost completely disappeared in DCC^–/–^ embryos (compare Figure 3E with 3F). Caudally, the *Pax7*^+^ population of the IPC was much reduced relative to that in the wild-type brains (compare Figure 3B with 3G). However, there were a few *Pax7*^+^ neurons that succeeded in reaching their final destination in the IPC (Figure 3G). Hence, the migration of *Pax7*^+^ cells into the IPR is more strongly affected than that into the IPC. On the other hand, there appear to be dense ectopic clusters of *Pax7*^+^ cells within both the r1-r and r1-c (yellow arrows in Figures 3F,G) that might correspond to cells arrested in their migration, as indicated above.

In the DCC^–/–^ embryos there appeared to be a reduced group of *Otx2*^+^ cells that reach their destination in the IPC. However, a large part of the *Otx2*^+^ did not reach the IPC at all, which was an incomplete structure in these embryos when compared to the wild-type brains (Figure 3C). Consistent with this disrupted migration, we observed a dense mass of ectopic *Otx2* cells arrested in the r1-c mantle layer (yellow arrows in Figure 3D).

By analyzing the migration of these cell populations to the IPN, we conclude that there is a general but differential impairment of their migration, such that migration of the Pro and IPR populations (*Nkx6.1*, *Irx2*, *Otp*, *Pax7*) was more compromised than that of the IPC cells (*Pax7*, *Otx2*).

## Discussion

In this study, we first reveal that the Netrin-1 receptor DCC is expressed extensively in the neurons forming the three rostrocaudal portions of the developing IPN (Pro, IPR and IPC) (Figure 4A). When we analyzed the migration of five molecularly identified neuronal populations of the IPN in DCC^–/–^ mice (*Nkx6.1*^+^, *Pax7*^+^, *Irx2*^+^, *Otp*^+^, and *Otx2*^+^ cells) we found that, as expected, the absence of this Netrin-1 receptor severely disrupts the migration of these diverse elements into the three portions of the IPN (Figures 4C–H). In these conditions, the IPN populations are born and specified but their migration is impaired, which hinders the formation of a normally constituted IPN complex.

Our earlier studies demonstrated that the migration of various neuronal populations into the IPN is crucial for the development of this nucleus (Lorente-Cánovas et al., 2012). These neuronal populations have different dorsoventral origins (*Pax7* and *Irx2* in the alar plate, *Otp* across the alar and basal plates, *Nkx6.1* and *Otx2* in the basal plate) and different rostrocaudal fates within the IPN (*Nkx6.1* and *Irx2* in the Pro; *Pax7*, *Otp* and *Irx2* in the IPR; and *Pax7* and *Otx2* in the IPC). These features of the different regions reflect the variant molecular profiles of the three neuromeric units that form the IPN complex, the Ist, r1-r and r1-c units, the latter two behaving histogenetically as independent neuromeres (Aroca and Puelles, 2005; Alonso et al., 2013).

Remarkably, the anteroposterior extent of the IPN is restricted to the rostral hindbrain area under the influence of the isthmic organizer (Crossley et al., 1996), that is thought to stop at the r1/r2 boundary (Aroca and Puelles, 2005). The rostrocaudal gradient of efficient FGF8 signaling delivered through the Ist and r1 (Aroca and Puelles, 2005) possibly accounts for part of the segmental differences in migration behavior and targeting, either directly or indirectly. Irrespective of such anteroposterior segmental differences, the Netrin-1/DCC pathway clearly emerges as a general shared mechanism that is crucial for the normal targeting of diverse cell populations to the plurineuromeric ventral subpial IPN complex.

Notably, several of the IPN cell populations studied have their origins in the lateral part of the basal plate, or even in the neighboring alar plate, such that they first have to migrate tangentially within the periventricular stratum to approach the paramedian region, where their final radial migration into the IPN occurs, adjacent to the local floor plate (Lorente-Cánovas et al., 2012). Our study shows that the absence of DCC affects both the tangential and radial migrations of the IPN cells. Nevertheless, some of them manage to migrate tangentially down to the paramedian region, failing thereafter in their final radial migration, while only a few are able to reach the IPN locus. This ability of some DCC-deficient cells to migrate suggests that additional mechanisms other than the Netrin-1/DCC pathway also influence these tangential and radial migrations.

In terms of the involvement of the Netrin-1/DCC pathway in IPN development, it would appear to first participate in the tangential dorsoventral migration of DCC^+^ cells, such that they would be attracted by the floorplate source of Netrin-1, in accordance with their known attractive interaction (Finci et al., 2015). The subsequent radial migration of these cells could be mediated by the attractive role of a second accumulation of Netrin-1 protein in the pial surface of the hindbrain paramedian region (Yung et al., 2018). Alternatively, the floor plate Netrin-1 (Yung et al., 2018; present data) could become a repulsive cue in collaboration with the Unc5 receptors (Hong et al., 1999) that are expressed by the developing IPN (van den Heuvel et al., 2013). Either of these two mechanisms (attractive sub-pial Netrin-1 or repulsive floorplate Netrin-1) could cause the radial migration of DCC^+^ cells toward their final sub-pial location after they have completed their tangential migration.

There are many homeodomain transcription factors that regulate tangential migration (Chédotal and Rijli, 2009). For example, concerning the transcription factors expressed in the IPN, *Nkx6.1* is involved in the tangential migration of branchiomotor neurons (Müller et al., 2003), while *Otp* is involved in the migration of diencephalic dopaminergic neurons (Ryu et al., 2007; Löhr et al., 2009) and some hypothalamic neurons (Acampora et al., 1999; Blechman et al., 2007). In terms of the IPN complex, we know that the transcription factor Otx2 is needed for the tangential migration of the *Otx2*^+^ cells of the IPC within the r1-c medial basal plate, as shown in knock-out mice (Ruiz-Reig et al., 2019). In this model, *Otx2*^–/–^ cells down-regulate their DCC expression, leading to the proposal that the Netrin-1/DCC pathway might participate in the tangential migration of this neuronal population.

On the other hand, the role of the floor plate derived morphogen SHH in the migration of the IPN populations was also investigated (Moreno-Bravo et al., 2014). SHH is a well-known organizer that participates in the formation of various ventral neuronal cell types (Placzek and Briscoe, 2005). In a *Shh*-conditional mutant (Moreno-Bravo et al., 2014), the basal plate is smaller and the basal *Nkx6.1* population disappears at the interpeduncular Pro portion. However, the alar *Pax7* population migrates ventrally possibly due to remnant Netrin-1 protein expressed in the floorplate of this mutant.

Although Netrin-1 has already been proposed as a potential regulator of migratory targeting to the IPN (Aroca et al., 2006; Moreno-Bravo et al., 2014; Ruiz-Reig et al., 2019), this issue had not been directly addressed to date. Our results indicate that along with the previously demonstrated involvement of *Otx2* (Ruiz-Reig et al., 2019) and SHH (Moreno-Bravo et al., 2014), the Netrin-1/DCC signaling system fulfils an important role in the independent IPN migrations examined. Regarding the molecular identity of the 5 groups of migrating IPN cells identified, it would be necessary to identify additional transcription factors, as well as other extra- and intracellular signaling molecules involved in cell migration.

In the absence of DCC, ectopic cells that express each of the markers analyzed accumulate in the basal mantle zone. These apparently correspond to cells whose migration is arrested or misdirected due to their inability to respond to the source of Netrin-1, provided either by the floor plate or by the paramedian sub-pial surface (see above). The different morphology and distribution of the ectopic cell clusters observed for each studied gene might be related to the respective molecular identities and places of origin. The presence of these dense ectopic groups of cells suggests that cell survival has not been affected in the absence of DCC and indeed, the loss of DCC does not normally produce cell death in other experimental models, consistent with the proapoptotic role of this molecule (Mehlen et al., 1998). Moreover, Netrin-1 has been shown to act as a survival factor (Llambi et al., 2001), which could explain the survival of IPN neurons despite residing in abnormal ectopic environments.

Overall, the data presented here, in conjunction with the known roles of the mHb-IPN system, indicate that the loss of Netrin-1/DCC signaling probably leads to multiple malformations in the IPN, which would be expected to cause severe brain dysfunction. In fact, lesion or genetic/pharmacological manipulation of the mHb-IPN system in experimental models leads to dysfunctional brain syndromes like depression (Xu et al., 2018), schizophrenia (Lecourtier et al., 2004), and substance use (reviewed by McLaughlin et al., 2017).

We assume that more subtle (or selective) deficits or variations of this multiple migrational mechanism might be caused by given DCC genetic variants. This may help explain the association of different genetic variants of Netrin-1 and DCC with depression, schizophrenia or substance use (Vosberg et al., 2020). This association of Netrin-1/DCC with certain psychiatric disorders can be related to the expression of these factors in the ventral tegmental area, nucleus accumbens and orbitofrontal cortex, all part of the limbic system (Flores et al., 2005; Grant et al., 2007, 2014; Flores, 2011; Vosberg et al., 2020). Interestingly, and as inferred from our results, the emergence of such psychiatric disorders could also be associated with the effects of pathogenic variants of DCC leading to IPN maldevelopment, especially considering that this nucleus is also a limbic structure connected to the ventral tegmental area (Groenewegen et al., 1986; Klemm, 2004; McLaughlin et al., 2017).

## Conclusion

We demonstrate that the Netrin-1/DCC pathway is involved in the migration of the IPN. We have not quantified the differences between the studied conditions from a statistical point of view. However, the qualitative results we show regarding deficits in cell migration are sufficient for our purpose, since we are not proposing any quantitative conclusions, nor a hypothesis that implies quantitative postulates. As such, we have not considered it necessary to perform a quantitative and/or statistical analysis to support the conclusions of this work. Our finding is important not only to understand the development of this structure, but also as a basis to study the possible neuroembryological etiology of brain disorders related to the habenulo-interpeduncular system. Further studies will be needed to ascertain experimentally if the known genetic *Dcc* variants also affect the migration of the different IPN populations, and eventually to analyze the resulting cellular and molecular features as well as the connectivity of this nucleus in these experimental conditions. It would be also interesting to discern additional cellular or molecular mechanisms regulating IPN migration together with Netrin-1/DCC.

## Data Availability Statement

The original contributions presented in the study are included in the article, further inquiries can be directed to the corresponding author.

## Ethics Statement

The animal study was reviewed and approved by Ethics Committee of the University of Murcia.

## Author Contributions

PA conceived and designed the research. IG-G and PA performed the experiments. IG-G, FM, and PA analyzed the data. AA and NM-D collaborated in the probe synthesis, image analysis, and figure preparation. BA took care of the transgenic mice colony, obtaining and genotyping the embryos. GL-B established and maintained the transgenic mouse colony at her institution. GL-B and LP provided funding for this study. IG-G, FM, and PA wrote the manuscript. IG-G, AA, NM-D, GL-B, LP, FM, and PA edited the manuscript. All the authors contributed to the article and approved the final version submitted.

## Conflict of Interest

The authors declare that the research was conducted in the absence of any commercial or financial relationships that could be construed as a potential conflict of interest.

## Abbreviations

IPN: interpeduncular nucleus
Pro: prodromal
IPR: interpeduncular rostral
IPC: interpeduncular caudal
Ist: isthmus
r1: rhombomere 1
r1-r: rhombomere 1 rostral
r1-c: rhombomere 1 caudal
r2: rhombomere 2
mHb: medial habenula
VZ: ventricular zone.
